# The ModFOLD4 server for the quality assessment of 3D protein models

**DOI:** 10.1093/nar/gkt294

**Published:** 2013-04-25

**Authors:** Liam J. McGuffin, Maria T. Buenavista, Daniel B. Roche

**Affiliations:** ^1^School of Biological Sciences, University of Reading, Reading, RG6 6AS, UK, ^2^BioComputing Section, Medical Research Council Harwell, Harwell Oxford, Oxfordshire, OX11 0RD, UK, ^3^Beamline B23, Diamond Light Source, Didcot, OX11 0QX, UK, ^4^Laboratoire de génomique et biochimie du métabolisme, Genoscope, Institut de Génomique, Commissariat à l'Energie Atomique et aux Energies Alternatives, Evry, Essonne 91057, France, ^5^UMR 8030 – Génomique Métabolique, Centre National de la Recherche Scientifique, Evry, Essonne 91057, France, ^6^Départment de Biologie, Université d’Evry-Val-d’Essonne, Evry, Essonne 91000, France and ^7^PRES UniverSud Paris, Saint-Aubin, Essonne 91190, France

## Abstract

Once you have generated a 3D model of a protein, how do you know whether it bears any resemblance to the actual structure? To determine the usefulness of 3D models of proteins, they must be assessed in terms of their quality by methods that predict their similarity to the native structure. The ModFOLD4 server is the latest version of our leading independent server for the estimation of both the global and local (per-residue) quality of 3D protein models. The server produces both machine readable and graphical output, providing users with intuitive visual reports on the quality of predicted protein tertiary structures. The ModFOLD4 server is freely available to all at: http://www.reading.ac.uk/bioinf/ModFOLD/.

## INTRODUCTION

Presently, protein structure prediction is the only viable means of closing the knowledge gap between protein sequences and their tertiary structures. Accurately predicted structures can be informative and useful indicators of function, but once you have generated a 3D model of a protein, then how do you determine its accuracy without the availability of the native structure? The ability to evaluate the quality of 3D models is not only important for the credibility of the field of structure prediction but also crucial for wet laboratory researchers to know whether they can trust a particular model (or regions of a model) to inform experimental work. During the past 20 years, the Critical Assessment of Techniques for Protein Structure Prediction (CASP) experiment has allowed us to determine some of the best methods for structure prediction; however, best methods do not always produce the best models; therefore, an estimation of the likely errors for any given model is crucial. After all, how much use would BLAST (1) be without an *E*-value? The ModFOLD4 server is the latest version of our popular independent server for the prediction of the global and local quality of 3D protein models. The server allows researchers to make value judgements about the credibility of models through the provision of accurate Quality Assessment (QA) data and intuitive graphical visualisations.

As the first models of protein structures were made researchers have developed tools to determine their quality. Early tools focused on basic stereochemical checks such as WHAT-CHECK (2), PROCHECK (3) and, more recently, MolProbity (4). Although these are good tools for providing a useful ‘reality check’ and identifying unusual geometric features in models, they do not produce single scores that allow you to rank numerous alternative models. Traditionally, statistically derived energy functions, such as ProSA (5) and DFIRE (6), have been used along with the alternative knowledge-based approaches, such as VERIFY3D (7), to provide single scores that relate to the global quality of protein models. In the past decade, machine learning-based QA programs, such as early versions of ProQ (8), QMEAN (9) and ModFOLD (10), have been used to increase the accuracy of predicted global model quality, using various combinations of structural features and/or a consensus of individual energy potentials.

Each of these methods can be categorised as true single-model-based approaches; in other words, they consider each model in isolation when making a calculation of global quality. However, as the QA category was introduced in the CASP7 (The 7th Community-Wide Experiment on the CASP), it has been clear that the so-called clustering approaches, which are based on the structural comparison of pools of multiple models, were often found to be superior to the traditional single-model-based approaches. Most of the successful clustering methods borrow from the 3D-Jury approach (11) where all-against-all structural comparisons are made to obtain mean similarity scores for ranking models. In recent CASP experiments (CASP8–CASP10), the clustering based approaches, such as the MULTICOM (12), Pcons (13), QMEANclust (14) and ModFOLDclust (15) method variants, have been consistent top performers in the QA category (16,17). However, the CASP8 and CASP9 assessments have been criticized for disadvantaging single-model methods and quasi-single-model methods in comparison with clustering methods, owing to availability of large sets of models (17). Clustering approaches have also been criticized for not addressing the real life needs of researchers; often researchers will want to evaluate a single model, or relatively few models, and in these cases, clustering methods will perform poorly (10). Such criticisms prompted a change of focus at CASP10 to rebalance the QA assessment using smaller bespoke data sets. The ModFOLD4 server was independently benchmarked at CASP10 and was found to be among the top performing methods in the QA category. The ModFOLD4 server can make use of the availability of large data sets containing hundreds of models, or it can evaluate single models with comparable performance, thereby providing accurate evaluations of model quality while addressing the real-life needs of researchers investigating protein structures.

## MATERIALS AND METHODS

**Figure 1.**
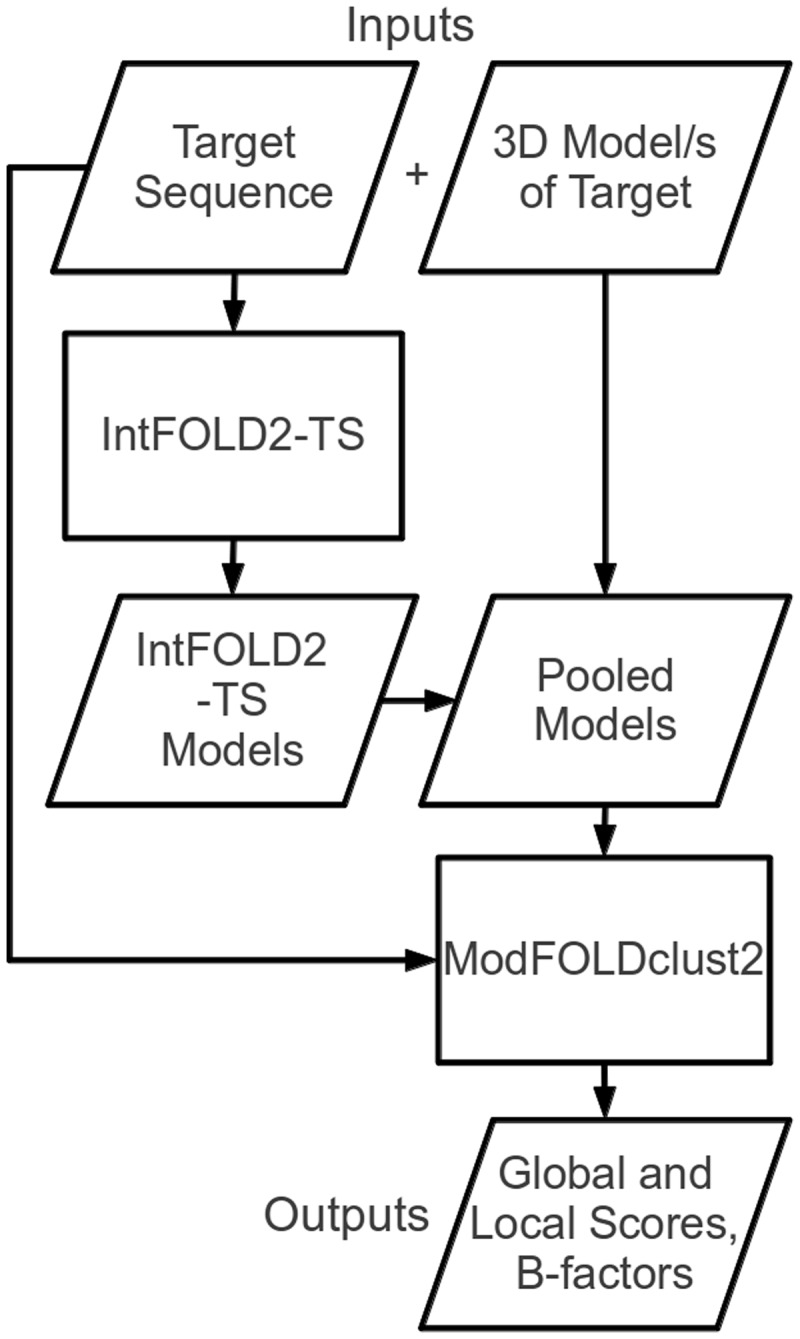
Flow chart outlining the principal stages of the ModFOLD4 server prediction pipeline.

The ModFOLD4 server deploys a quasi-single-model QA algorithm. This means that the method preserves the predictive power of pure clustering-based methods while also being capable of making predictions for a single model at a time. If the server receives multiple models then it will make use of a full clustering approach; however, if only a single model is submitted, then it will operate in quasi-single-model mode with comparable accuracy (see ‘Results’ section). Figure 1 shows a simplified flow chart outlining the principal steps of the ModFOLD4 server prediction pipeline. The target sequence and 3D model (or multiple 3D models) of the target are submitted via the web submission form (http://www.reading.ac.uk/bioinf/ModFOLD/ModFOLD_form_4_0.html). The target sequence is then processed via the IntFOLD2 tertiary structure prediction pipeline (IntFOLD2-TS) (18,19).

The IntFOLD2-TS protocol generates multiple template models using a quality filtering mechanism for the selection of multiple sequence-structure alignments (18). The protocol comprises three main stages. In the first stage, the method generates 90 initial single template models (10 models each from 9 alternative alignment methods). In the second stage, the 90 single-template models are clustered using the ModFOLDclust2 method (20) to obtain initial global and local quality scores. In the third stage, ∼80 multi-template models are generated by selecting from the pool of initial alignments on the basis of both global and local quality scores. Thus, the initial alignments are re-ranked and screened according to quality, and any overlapping regions from multiple templates are only included if the predicted local quality is predicted to be improved.

The final ∼80 alternative multi-template models produced by IntFOLD2-TS for the protein target are used to gauge the relative quality of each 3D model submitted by the user. If multiple models have been submitted by the user, then all models are pooled for the comparison. The pool of submitted models and IntFOLD2-TS models are then evaluated using the ModFOLDclust2 method (the target sequence is also used at this stage for the correct numbering of residues) (20).

The ModFOLDclust2 method is based on the old 3D-Jury approach (11) of pairwise structural comparisons of multiple models, often referred to as clustering. In ModFOLDclust2, the global scores are calculated using the combination of a structural alignment independent scoring method (ModFOLDclustQ) and the original ModFOLDclust method (10) that exploits the TM-score (21), thereby increasing prediction accuracy with minimal computational overhead (20). From the global scores of models, we can calculate *P*-values to represent the probabilities that each model is incorrect. To calibrate the *P*-values, we used a similar approach to that previously adopted for measuring the coverage of genomic scale fold recognition (22,23). The server models for the CASP7, CASP8 and CASP9 targets were downloaded (http://www.predictioncenter.org/download_area/), and the predicted global quality scores for each model were calculated. The observed quality scores of models were then calculated, using TM score to compare models with the native structures, and models with TM scores <0.2 were taken to be incorrect (24). The predicted scores from the pool of incorrect models provided a score distribution to which we fitted a density curve. We were then able to determine the statistical significance of any score using this curve. Therefore, for any given global model quality score, a *P*-value can be provided that represents the proportion of models that do not share any similarity with the native structure.

The local model quality is evaluated by using a score similar to the average *S*-score, which has previously been used for model evaluation in servers such as Pcons (13) and the original version of ModFOLDclust (15). For a residue in a pairwise superposition the *S*-score is defined as:

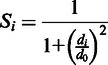



where *S_i_* is the *S*-score for residue *i* in a model, *d_i_* is the distance between aligned residues according to the TM-score superposition and *d_0_* is the distance threshold (3.9). An *S_i_* score of 0 is given if *d_i_*> 3.9 Å. The *S*-scores for each residue are summed, and the mean score is calculated:

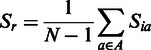



where *S_r_* is the predicted residue accuracy for the model, *N* is the number of models for the target, *A* is the set of alignments and *S_ia_* is the *S_i_* score for a residue in a structural alignment (*a*). The size of set *A* is equal to *N*-1. The mean *S*-score for each residue is then converted to the predicted distance from the equivalent residue in the native structure (*d_r_*), by simply rearranging the equation for the *S*-score:

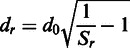



An upper limit of 15 Å is set for *d_r_*. Missing residues in the model are represented by an ‘X’ in the prediction.

All of the models in the pool are ranked by global model quality score, and a *P*-value is calculated for each, which relates to the likelihood that the global model is incorrect. Local quality score information is also provided in graphical and machine-readable formats. The predicted distances of each residue in the model from the equivalent residues in the native structure (Ångströms) are also added into the B-factor column of each submitted model.

## RESULTS

### Independent blind testing at CASP10

For CASP10, we participated with three variants of the ModFOLD method, the pure clustering approach ModFOLDclust2 (Group 423), the default ModFOLD4 server (Group 366) and the ModFOLD4_single server (Group 196), which operated in forced single-model mode so that each model was considered in isolation. ModFOLD4_single allowed us to determine the accuracy of the method if users were to submit one model at a time. The performance of the ModFOLD4 server is found to be comparable to pure clustering methods, both in terms of global (QMODE1, QA1) and local QA (QMODE2, QA2). The results from the official assessment are summarized in http://predictioncenter.org/casp10/doc/presentations/CASP10_QA_AK.pdf. For an interactive detailed comparison of methods and scores see http://predictioncenter.org/casp10/qa_analysis.cgi.

### Server inputs and outputs

#### Inputs

The only required inputs to the server are the amino acid sequence for the target protein (on which the model is based) and a single 3D model [in Protein Data Bank format] for evaluation. However, users may optionally upload the following: multiple alternative models (as a tarred and gzipped directory of PDB files), a name for their protein sequence and their email address. The time taken for a prediction to complete will depend on the length of the sequence, the number of models submitted and the load on the server (20). Typically, users should expect to receive results back for a single model within a few hours and certainly within the same day. If several hundred models have been submitted for a single target, then it may take several days before the results are returned.

#### Graphical outputs

**Figure 2.**
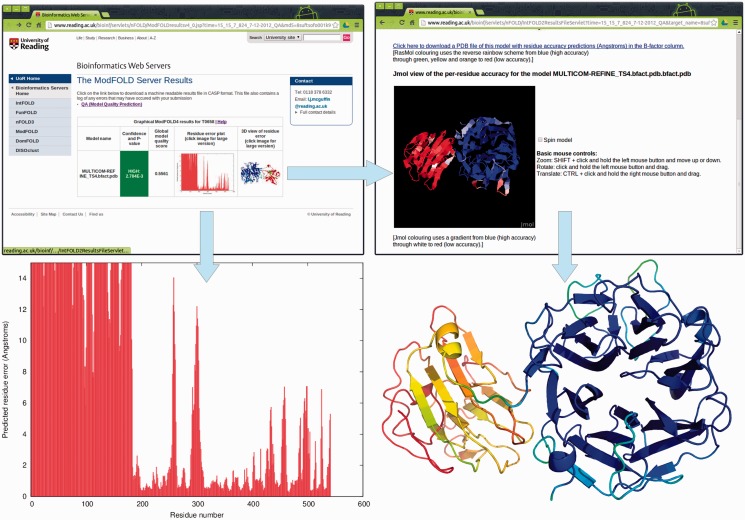
Graphical and interactive output from the ModFOLD4 server. Top left: main results page with table showing global scores, *P*-values and thumbnails of graphical output (results for a single model are shown for clarity; multiple models are shown as additional rows to the table—see the examples on the help pages). Bottom left: clicking on the thumbnail image leads to a full size view of the per-residue error plot, which may be downloaded in PostScript format. Top right: clicking on the thumbnail image leads to an interactive view of model, which can be manipulated in 3D using the Jmol plug-in (http://jmol.sourceforge.net/). Bottom right: PDB files of the model can be downloaded with local quality scores added to B-factor column. The model can be viewed and rendered in PyMol (http://www.pymol.org/) using the temperature colouring option, where blues and greens represent residues predicted to be close to the native structure, whereas oranges and reds represent those that deviate from the native structure.

The server provides a clean and simple interface so that results may be viewed on a single page and easily interpreted by non-experts at a glance. The types of graphical output that are provided by the server are illustrated in Figure 2. The results page consists of a single table summarizing the QA scores for each submitted model. Each row in the table includes the following: a global score for the model, a *P*-value indicating the likelihood that the global model is incorrect and a plot of the local errors in the model (the predicted distance in Ångströms of each residue from the native structure). Thumbnail images link to new pages providing more details about the quality of the local regions of the model.

#### Predicted B-factors

Conveniently, the server also inserts the predicted local quality scores into the B-factor column of the ATOM records for each submitted model. The results table includes a graphical view of each model coloured by predicted B-factors or temperature scheme. Additionally, users may interactively manipulate annotated models in 3D using the Jmol plug-in. The B-factor scores added to models are highly accurate [the area under curve (AUC) is ∼0.9 according to independent evaluations: http://predictioncenter.org/casp10/doc/presentations/CASP10_QA_AK.pdf].

#### Machine readable outputs

Two main types of machine readable files are created by the server and made available for download, primarily in the interest of developers, which comply with the CASP data standards (http://predictioncenter.org/casp10/index.cgi?page=format#QA). First, the QMODE2 (QA2) formatted results file is available to download, containing raw global and local quality scores for each submitted model. Second, the PDB files for each uploaded model may be downloaded with the predicted local quality scores added to B-factor column.

## CONCLUSIONS

The ModFOLD server has been used extensively by researchers worldwide during the past 5 years, and it remains one of the most accurate servers for the QA of 3D models of proteins. The original article describing the ModFOLD server was published in 2008 (25), but it has since undergone a number of major updates and repeated independent testing during successive CASP experiments. The latest server implementation of ModFOLD (version 4.0) includes an improved quasi-single-model-based algorithm that is competitive with the best clustering-based methods. The ModFOLD4 server was recently independently assessed during CASP10 experiment using rigorous performance benchmarks, and it was found to rank among the top few methods internationally. The server is also a partner site of the protein model portal (26) (http://www.proteinmodelportal.org) for model quality estimation.

## FUNDING

Studentship from the University of Reading, MRC Harwell and the Diamond Light Source (to M.T.B.); European Union Seventh Framework Programme (FP7/2007-2013) [246556 to D.B.R.]. Funding for open access charge: University of Reading; MRC Harwell; Diamond Light Source.

*Conflict of interest statement*. None declared.
